# Focal Muscle Vibration and Physical Exercise in Postmastectomy Recovery: An Explorative Study

**DOI:** 10.1155/2017/7302892

**Published:** 2017-03-26

**Authors:** Claudia Celletti, Maria Antonietta Fara, Guido Maria Filippi, Giuseppe La Torre, Roberto Tozzi, Nicola Vanacore, Filippo Camerota

**Affiliations:** ^1^Physical Medicine and Rehabilitation Division, Umberto I Hospital, Rome, Italy; ^2^Rehabilitation and Recovery Division, P.O. Nord, Az. Usl Latina, Latina, Italy; ^3^Institute of Human Physiology, Catholic University, Rome, Italy; ^4^National Centre for Epidemiology, Surveillance and Health Promotion, National Institute of Health, Rome, Italy; ^5^Department of Public Health and Infectious Diseases, University Sapienza of Rome, Rome, Italy

## Abstract

*Background*. Physical activity initiation and maintenance are particular challenges in the postmastectomy recovery and in particular Dragon Boat racing seems to be a useful sport activity. The aim of this study was to evaluate the role of focal muscle vibration as a proprioceptive input to improve upper limb functioning in a group of “paddlers” patients.* Methods*. A group of paddlers has been evaluated before vibratory treatment (T0), immediately after therapy (T1), after one week (T2), and after one month (T3) with DASH questionnaire, Body Image Scale, McGill pain questionnaire, Constant Scale, and Short Form 36 questionnaire.* Results*. Fourteen patients showed a significant reduction in disability score (*p* = 0,001) using DASH scale, an improvement of upper limb function (*p* = 0,001) using the Constant scale, and a reduction of pain (*p* = 0,007) at the McGill pain questionnaire. The Mental Composite Score of the Short Form 36 questionnaire showed significant results (*p* = 0,04) while no significant results had been found regarding the physical mental score (*p* = 0,08).* Conclusion*. Focal muscle vibration may be a useful treatment in a postmastectomy recovery of upper limb functionality.

## 1. Introduction

Breast cancer is the second most common cancer in the world and the most frequent cancer among women with an estimated 1.67 million new cancer cases diagnosed in 2012 (25% of all cancers) [1].

Treatment of breast cancer can include surgery to the breast and axilla and adjuvant chemotherapy, radiotherapy, and endocrine therapies. These interventions have increased survival but can induce chronic side effects such as breast cancer-related lymphedema, upper body functional impairment, chronic fatigue, and psychological impairments (e.g., depression) [2].

Following surgery for breast cancer, women may experience substantial impairment in upper extremity function. Functional limitations, including decline in strength and range of motion, may continue after acute recovery from surgery is complete [3].

Physical activity initiation, reinitiation, and maintenance are particular challenges in the posttreatment population. Authorities from the Institute of Medicine (IOM), American Cancer Society, American College of Sports Medicine, and others [4, 5] have recommended physical activity (PA) as a critical, a safe, and an effective part of survivorship planning. Specifically, a meta-analysis found that exercise is associated with improved QoL, physical functioning, and fitness and reduced fatigue in breast cancer patients and survivors [6].

Furthermore a number of growing studies have been focused on body image issues in cancer, such as among BC patients. In fact, as well as functional and muscle limitations, patients with BC aftereffect show a cognitive dynamic perception of physical appearance of their own body, until phantom symptoms [7], that may aggravate also the functional recovery. Recently Dragon Boat racing has been introduced for survivors as a resistance strenuous exercise for the upper body extremities and trunk, associated with a lower risk of injury, and studies demonstrated that the “paddlers” showed a marked improvement in both physical and mental health [8].

In a recent and relatively wide group of studies, a particular type of mechanical vibration, at high frequency (100 Hz) and low amplitude (0.2–0.5 mm), focally applied on selected muscles, showed significant improvement of endurance, strength, power, joint stiffness control, and body image perception [9–15]. These effects are likely due to a direct effect on the central nervous networks which control the motor execution [9–15]. The effects were observed both in healthy and in impaired individuals, including elderly people, characterized by asthenia.

According to these reports, we decided to explore the possibility of an improvement of the perception of body image and the functional improvement of upper limb in a group of skilled “paddlers” patients after a treatment with focal muscle vibration. The aim of this study was to explore a new possible approach in the improvement of upper limb function after postsurgical BC in order to better participate in the sport and activity and in improvement quality of life.

## 2. Materials and Methods

### 2.1. Patients

**Figure 1:**
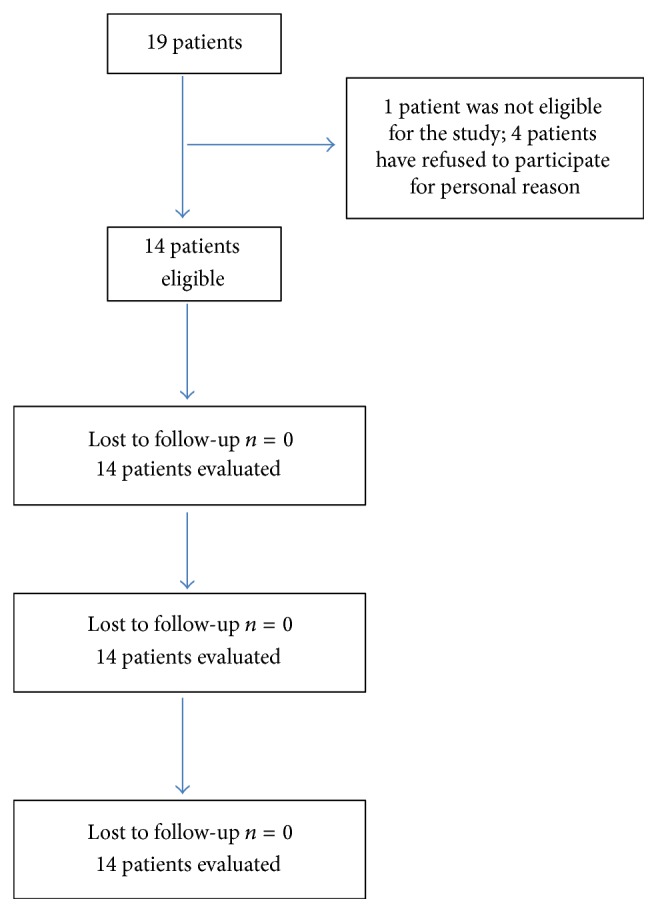
Flowchart.

Women following the service of “recovery and rehabilitation” of Latina Hospital had been recruited in this study; the flowchart of the trial is illustrated in Figure 1. Patients had been evaluated before vibratory treatment (T0) and immediately after therapy (T1) and after one week (T2) and one month (T3). All the patients were included in the Dragon Boat program and they were playing paddler from at least 6 months before the recruitment in this study.

Including criteria were breast cancer surgically treated at least 12 months before with upper limb aftereffects, in particular functional limitation of shoulder associated or not with modest-moderate upper limb lymphedema. Exclusion criteria were active phase of breast cancer or other neoplastic pathologies, ongoing radio- or chemotherapy, severe shoulder range of motion limitation (less than 20° of flexion and shoulder abduction), severe or inveterate lymphedema, concurrent lymphangitis or mastitis, and other neurological or orthopedics conditions that may influence upper limb functionality.

The study was designed in accordance with the Declaration of Helsinki (1964) and approved by the local ethics committee (study protocol Lazio 2 SL55-13). All study participants provided informed consent.

### 2.2. Outcome

All patients have been clinically evaluated measuring upper limb circumference (over and under elbow and wrist), joint range of motion, and muscle strength.

Furthermore, patients have been evaluated using the following scales: DASH questionnaire, Body Image Scale, McGill pain questionnaire, and Constant Scale. Health-related quality-of-life measures were obtained through a patient-oriented tool: Short Form 36 questionnaire (SF-36).

The DASH questionnaire is a regional outcome measure suitable for patients with upper extremity musculoskeletal conditions and consists of 30 items [16]. Each item is scored on a 5-point scale. The scores for all items are used to calculate a final score ranging from 0 (no disability) to 100 (severe disability). The average time taken by patients to complete the questionnaire is 6 min. Every item contained in the DASH requires the use of the upper extremity. Reliability, validity, and responsiveness of the DASH have been evaluated in patients with disorders of all major areas of the extremity, that is, shoulder, elbow, wrist, and hand. The DASH is well correlated with most of the dimensions of the SF-36 and is considered a valid measure of health status.

Body Image Scale (BIS) is a 10-item short scale for use in clinical trials to evaluate body image as an important endpoint in quality-of-life evaluation since cancer treatment [17].

The McGill pain questionnaire [18] is probably the most well-known and complete tool for the verbal assessment of pain. It provides a subjective measurement of pain intensity as well as clues on qualitative features of the chronic or acute pain experienced by the patients. Results broadly fall into 3 main classes: (1) sensory qualities (temporal, spatial, thermal, pressure, and other qualities); (2) affective qualities (tension, fear, and autonomic properties); (3) intensity of pain. Descriptors are also subdivided into 20 subclasses and arranged in increasing order of pain intensity.

The Constant–Murley Score (CMS) is the most commonly used outcome tool containing objective measurement and has been found to be valid and reliable for a range of shoulder pathologies, including rotator cuff disease [19, 20]. CMS includes both self-reported and performance-based items. Score can vary between 0 and 100: a minimum total score of 80 points is considered in general a good result for the patient (i.e., patient recovered).

SF-36 consists of 36 questions that inquire about the general health status of patients. This questionnaire provides eight specific categories of physical and emotional scores (Physical Functioning, PF; Role Physical, RP; Bodily Pain; General Health; Vitality; Social Functioning; Role Emotional, RE; Mental Health), resumed on two main scores: Physical Composite Score and Mental Composite Score. Very low scores for the Physical Composite Score indicate severe physical dysfunction, distressful bodily pain, frequent tiredness, and unfavorable evaluation of the health status. Very low scores for Mental Composite Score indicate frequent psychological distress and severe social and role disability due to emotional problems [21]. The official Italian version [22] was used; administration to the patients was performed in agreement with standardized methodologies [22–24].

### 2.3. Intervention

Vibratory stimulation was applied according the same protocol applied in other studies [9–15] to the muscles using a specific device that consisted of an electromechanical transducer, a mechanical support, and an electronic control. The support was rigidly anchored to the floor to guarantee good mechanical contact with tissue. A mechanical arm permitted the transducer to be placed on the treatment site. The soft tissues were compressed to ensure better transmission of vibrations to the muscles. The transducer was perpendicularly applied to the muscle, near its distal tendon insertion, and generated a sinusoidal displacement of 0.2 to 0.5 mm (peak to peak). The transducer was driven to produce forces ranging between 7 and 9 N. The vibration frequency was set at 100 Hz.

**Figure 2:**
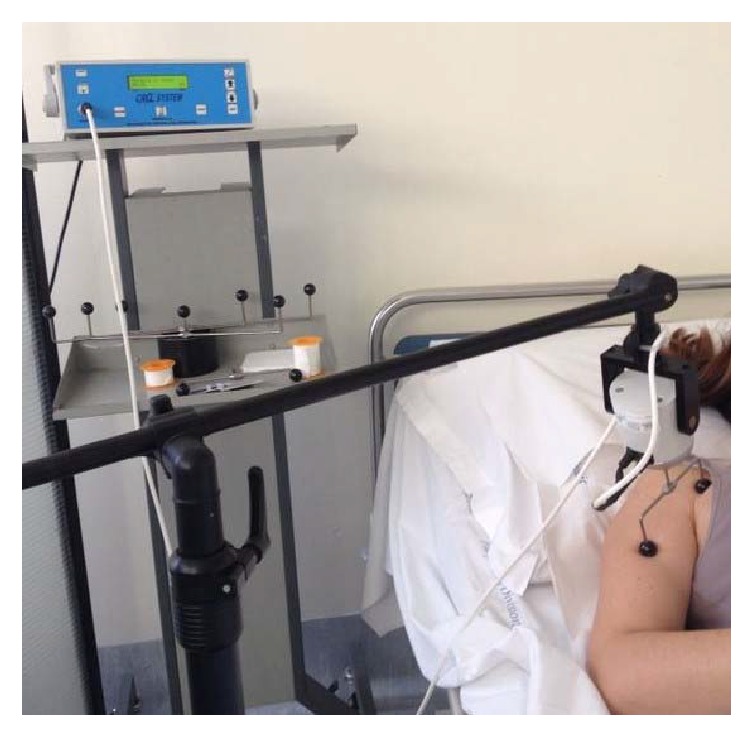
Upper limb application of focal muscle vibration.

During muscle vibration, the participants were supine, and they were requested to develop an isometric contraction of the treated muscles. The assessors monitored the muscular contraction throughout the series of applications; this procedure too is the same as described and detailed in the other studies [9, 10]. Figure 1 shows a typical treatment setting. Focal vibration was applied to the pectoralis* minor* and the* biceps brachii* of the affected limb (Figure 2). The mechanical applications were applied over 3 consecutive days. Every application consisted of 3 vibration sessions, each with a duration of 10 minutes. A 1-minute interval separated the sessions. During the intervals, stimulation was interrupted and the subject was requested to relax the muscle.

### 2.4. Dragon Boat

Dragon boating is a strenuous, repetitive upper body exercise, as 18 up to 22 team-mates propel a 12-meter long boat through the water that projects a visible message to all people with breast cancer [25]. It uses predominantly upper extremity and trunk muscles, and the improvement in strength has a carry-over effect to day-to-day activity. The training intensity can be varied simply by pulling harder. In many ways, it is an ideal exercise. It is non-weight-bearing and therefore is associated with a lower risk of injury than weight-dependent activities such as running. It is safe, and with proper technique the paddler can recruit a reasonable amount of muscle mass and induce positive adaptations in the musculoskeletal and cardiovascular systems [26]. All the patients have constantly participated to the Dragon Boat activity two times a week from at least 10 months before (median time 40 months before the study).

### 2.5. Statistical Analysis

The statistical analysis was conducted with the SPSS software package for Windows. The statistical analysis of the continuous variables was conducted calculating median and range (min–max), because these variables were not normally distributed. To evaluate the changes of the DASH scale, Body Image Scale, and McGill pain questionnaire between the T0 to T1, T2, and T3 we used the Friedman test for paired samples. The probability level for statistical significance in all tests was set at *p* < 0,05.

## 3. Results

Five patients were not eligible for the study; one patient was excluded for ongoing acute pathology and four patients refuse to participate. 14 patients (median age 53,5 years) with chronic posteffect of mastectomy (median time from the surgical event 84 months min 12–max 192) were finally considered eligible for the study (demographic characteristics are shown in Table 1).

The clinical evaluation of upper limb circumference showed no statistical significant difference before and after one month at the elbow (*p* = 0,36), 10 cm above elbow (*p* = 0,40), 10 cm under elbow (*p* = 0,28), and at wrist (*p* = 0,73).

Evaluating the DASH score we have observed a significant reduction of the global score at T1, T2, and T3, with a progressive reduction along the all period (Table 2).

Body Image Scale shows a significant difference only between the evaluation at the end of the follow-up in comparison to the initial evaluation (Table 2).

Concerning pain intensity McGill pain questionnaire (Table 2) showed a reduction of pain intensity in the global score and also in the affective and evaluative subcomponents, which resulted in statistically significant reduction in all the evaluation timing (T1).

**Figure 3:**
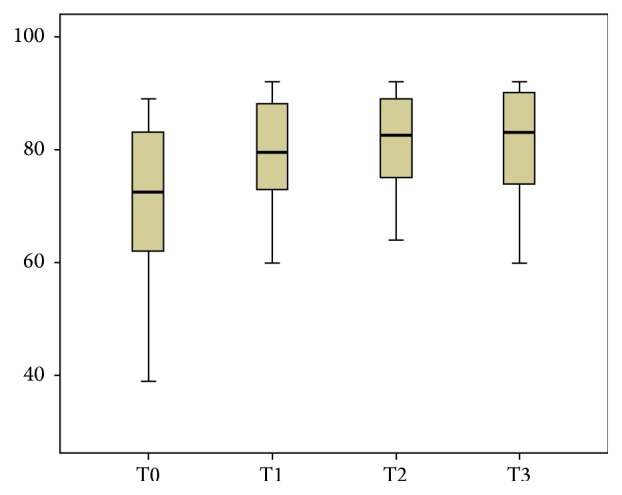
The box plot diagram of the Constant Scale.

About performance and shoulder functioning, also the Constant Scale score showed a significant increase in all the time evaluation, with an improvement of the total score and of the subitems. Interestingly the median score before the treatment was lower than value considered in general as a good result functional outcome for the patient after a shoulder injury; this score is exceeded at the follow-up evaluation (Figure 3).

Globally the perception of both physical functioning and mental component seems to be improved with a statistical amelioration of the Mental Composite Score (Table 3).

## 4. Discussion

The study evidenced a significant improvement of DASH scale and of the pain. Large part of such benefits was already significant at T1 and persisted at T2 and T3.

The first challenge is to understand the origin of such improvements. The role of the skilling in paddling, and consequently a progressive increase of the efficacy of the exercise, may be discarded since all the participants, at T0, were already playing paddler from at least 6 months. A second possibility, of course, may be the Dragon Boat exercise itself. However, it is improbable role of the exercise in both the intervals T0-T1 and T0–T2; actually the literature shows that physical training shows its efficacy at least after 12 weeks [26]. However at least at time T3 a contribution of the exercise cannot be excluded.

If a dominant role of the applied vibration may be suggested, it needs a discussion concerning the action modalities, in the light of the previous studies.

The time course of the effects is probably the primary aspect to debate. Important benefits are reported at the end of the 3 consecutive days of the intervention (T1). Such rapidity is commonly reported in the other studies based on the same protocol and the most probable reason of such time course is related to a direct action on the motor control nervous system [14]. In particular, the time course of this protocol was recently detailed [13].

A second aspect is the type of the improvements. Pain relief and ROM increase are coherent with many previous studies in which the same protocol was applied to reduce muscle hyperactivity in different diseases like stroke and children palsy [14, 27–29]; both these indexes are likely due to a reduction of the pectoral muscle spasm after mastectomy [30]. Pain relief and ROM increase may be supposed to have a role in the higher score in the quality of life of the participants. Quality of life is a complex parameter and is probably influenced by the body image. In particular, a percentage of patients after surgery may show a Phantom Breast syndrome [8], a set of symptoms phenomena normally associated with limb amputations; the syndrome involves pain at the site of the mastectomy alone or a simultaneous sensation of the presence of the breast while with sensation of pain tingling or heaviness is reported in isolation that needs to be treated in the rehabilitation program [8]. It was shown that this particular vibratory protocol significantly improves the body perception in the space, likely by an action on the sensitive compartment of our nervous system [12, 13].

## 5. Conclusion

This study has relevant limits in the small number of individuals, in its application at ≥12 months after the surgical procedure, on a group of women already trained. However, this study was built to explore a possible utility of the focal vibration in rehabilitation after mastectomy and the results, the short time application, and long lasting effects suggest that the protocol might have the potentialities to be useful treatment in a postmastectomy recovery of upper limb functionality, to improve quality of life and muscle performance, by favoring greater adhesion to physical activity.

## Figures and Tables

**Table 1:** Demographic characteristics of patients.

Age			Weight			Laterality		Cancer side		Type of surgery	
Median	Min	Max	Median	Min	Max	Right	Left	Right	Left	Mastectomy	Madden radical mastectomy
53,5	44	70	63,5	56	90	12/14	2/14	8/14	6/14	10/14	4/14

**Table 2:** The main outcome score evaluated before vibratory treatment (T0) and immediately after therapy (T1) and after one week (T2) and one month (T3). In bold *p* ≤ 0,05.

	T0	T1	T2	T3	T0/T3	T0-T1	T0–T2	T0–T3
DASH	54.2 (25–98.68)	50.32 (24.34–81.57)	47.69 (25.65–80.92)	39.14 (24.34–84.86)	**0,001**	**0,001**	**0,029**	**0,001**

Body Image Scale	12.5 (2–29)	9.5 (0–26)	8.5 (0–29)	8 (0–28)	0,091	0,13	0,096	**0,011**

McGill	37,5 (19–68)	36,5 (5–57)	32,5 (3–62)	29 (4–70)	**0,007**	0,052	**0,001**	**0,033**
Sensory	23,09 (11,33–28,71)	22,83 (1,7–28,6)	21,99 (0–27,61)	22,3 (2,35–30,78)	0,23	0,052	0,052	0,40
Affective	15,27 (2,42–17,71)	15,23 (0–17,01)	15,13 (0–17,1)	15,13 (0–17,62)	**0,003**	**0,021**	**0,021**	**0,007**
Evaluative	2,42 (1,89–4,42)	2,42 (0–4,42)	1,89 (0–3,76)	2,15 (0–4,42)	**0,016**	**0,034**	**0,02**	**0,034**
Mixed sensory	5,55 (0–7,46)	5,44 (0–6,98)	5,4 (0–7,4)	5,4 (0–7,4)	0,608	0,317	0,612	0,48
Mixed affective evaluative	2,97 (0–3,2)	2,72 (0–3,2)	2,25 (0–3,2)	2,25 (0–3,2)	0,30	1,00	0,56	0,18

Constant	72,5 (39–89)	79,5 (60–92)	82,5 (64–92)	83 (60–92)	**0,001**	**0,001**	**0,001**	**0,002**
*Pain subscore*	10 (0–15)	15 (10–15)	15 (5–15)	15 (5–15)	**0,010**	**0,008**	**0,014**	0,059
*AVQ subscore*	17 (12–20)	19 (15–20)	20 (15–20)	20 (15–20)	**0,006**	**0,02**	**0,02**	**0,011**
*ROM subscore*	39 (24–40)	40 (32–40)	40 (34–40)	40 (34–40)	**0,001**	**0,014**	**0,008**	**0,008**
*Strength subscore*	8 (3–14)	10,5 (3–17)	12 (2–17)	11 (1–17)	**0,007**	**0,011**	**0,004**	0,083

**Table 3:** SF-36 score before treatment and after one month. In bold *p* ≤ 0,05.

SF 36	T0	T3	*p* (T0–T3)
Physical Functioning (PF)	80 (45–100)	80 (45–100)	0,20
Role physical (RP)	0 (0–100)	87,5 (0–100)	**0,008**
Pain (BP)	41 (12–100)	67,5 (22–100)	**0,02**
General Health (GH)	64 (25–97)	64 (0–97)	1,0
Energy/Vitality (VT)	50 (15–90)	55 (25–80)	0,24
Social Functioning (SF)	50 (12–87)	68,5 (12–87)	**0,011**
Role mental (RE)	33 (0–100)	84 (0–100)	0,10
Mental Health (MH)	44 (24–72)	56 (28–88)	0,16
PCS	57,92 (32,14–76,42)	64,21 (60,71–79,28)	0,08
MCS	48,78 (37,92–49,71)	58,21 (52,14–63,92)	**0,04**
